# HIV transmission as a result of drug market violence: a case report

**DOI:** 10.1186/1752-1505-2-8

**Published:** 2008-07-18

**Authors:** Will Small, Thomas Kerr, Evan Wood

**Affiliations:** 1 British Columbia Centre for Excellence in HIV/AIDS, St. Paul's Hospital, 608-1081 Burrard Street, Vancouver, British Columbia, V6Z 1Y6, Canada; 2 Department of Medicine, 2194 Health Sciences Mall, University of British Columbia, Vancouver, British Columbia, V6T 1Z3, Canada

## Abstract

While unprotected sexual intercourse and the use of contaminated injection equipment account for the majority of HIV infections worldwide, other routes of HIV transmission have received less attention. We report on a case of HIV transmission attributable to illicit drug market violence involving a participant in a prospective cohort study of injection drug users. Data from a qualitative interview was used in addition to questionnaire data and nursing records to document an episode of violence which likely resulted in this individual acquiring HIV infection. The case report demonstrates that the dangers of drug market violence go beyond the immediate physical trauma associated with violent altercations to include the possibility for infectious disease transmission. The case highlights the need to consider antiretroviral post-exposure prophylaxis in cases of drug market violence presenting to the emergency room, as well strategies to reduce violence associated with street-based drug markets.

## Introduction

Human immunodeficiency virus (HIV) transmission among injection drug users (IDU) represents a significant factor driving the global HIV epidemic [1], and HIV incidence remains elevated among this population in numerous settings globally [2]. While the use of contaminated injection equipment and unprotected sexual intercourse account for the majority of infections worldwide, other routes of HIV transmission have received less attention. For instance, few studies have examined less common routes of HIV infection [3], and we know of none that have considered the potential of direct blood-to-blood contact via violent altercations among IDU. Although HIV infection through violent interaction is likely rare, the potential of antiretroviral post-exposure prophylaxis to reduce the risk of HIV transmission under these circumstances makes it important to examine this potential route of transmission among drug user populations [4,5]. This may be particularly important given the high rates of drug market violence among IDU [6].

## Case description

We present the circumstances surrounding the HIV infection of a 39 year old white male who is a participant within a prospective epidemiological cohort study of IDU in Vancouver, Canada. In addition to completing a quantitative survey which assesses HIV risk behavior, study participants also provide a blood sample for HIV testing at semi-annual visits. As well, the cohort has a concurrent qualitative component designed to examine HIV seroconversions among participants. Qualitative interviews are conducted with cohort participants who recently received a positive HIV test result, to collect additional data regarding sexual, and injection behavior in the time prior to seroconversion, as well as details regarding potential sources of infection. Data from qualitative interviews generate a detailed description of circumstances surrounding each individual's HIV infection and are triangulated with cohort data and nursing records from pre-test counselling.

The present case, to whom we have given the pseudonym 'Peter', was defrauded in the street drug market by an individual who had sold him "bunk" [counterfeit drugs] instead of $50 worth of heroin he sought to purchase. When he next encountered the person who had defrauded him at a focal point of the local drug market, Peter attempted to obtain re-imbursement of his $50. When re-imbursement was refused, a physical altercation ensued, and the man who defrauded Peter was severely beaten. The assault was committed using his fists and no biting was involved. Peter came into contact with large volumes of the other man's blood, and reported that the skin on his own hands was broken in a number of places as a result of punching the victim. Peter reported having been aware that the victim was possibly HIV infected and that his open wounds posed potential for infectious disease transmission. However, he did not seek medical attention due to his fear of being identified by police.

Early in 2006, approximately 3 months after the incident, Peter presented to our research office for his semi-annual HIV test. Reviews of this participant's pre-test counseling nursing records from this study visit revealed that he suspected that he had been exposed to the HIV virus through the incident detailed above. In the six months prior to his positive test result, the present case was injecting drugs but his questionnaire data revealed that he denied any injection-related HIV-risks as he primarily injected alone and did not share syringes or ancillary equipment. Similarly, while the present case was sexually active in the 6 months prior to his seroconversion, he reported consistently using condoms and did not report any unprotected sex.

## Discussion

We have described a case of an individual whose HIV infection appears to be attributable to blood-to-blood contact which occurred during a violent encounter. The assault that likely resulted in this infection was sparked by a conflict in the local street-based drug market. Although this individual recognized that he had come into contact with the blood of a person he thought to be HIV positive, and was cognizant that there was potential for infectious disease transmission, he did not seek medical assistance.

Although violent encounters, particularly fistfights, involving HIV positive individuals have previously been identified as a mode of HIV transmission [3,7,8], this potential route of transmission has not been commonly reported, and as far we know, has never been reported among IDU. The present case suggests that the potential for HIV exposure should be explored among individuals involved in violent encounters in high HIV prevalence settings, especially among IDU, since the level of violence involved in this case is not unique [9,10]. This episode indicates that the dangers of violence among IDU extend beyond the immediate physical trauma associated with violent altercations to include the possibility for infectious disease transmission. The potential for high levels of violence among IDU to create opportunities for HIV transmission merits consideration of measures to provide antiretroviral post-exposure prophylaxis to individuals who have been involved in a violent encounter involving direct blood contact.

Within illegal drug markets violence is endemic and is employed for the purposes of punishment and conflict resolution [9-11], as persons buying or selling drugs in these markets have no recourse to legitimate authority to resolve disputes [12,13]. Therefore, effective practical interventions are needed to reduce the prevalence of drug market violence and mediate the negative health impacts that result. Substitution therapies (e.g., methadone, heroin prescription) and strategies to regulate or decriminalize particular illicit substances may hold potential to reduce violence among drug users and community levels of violence in neighborhoods where street-based drug markets currently operate [14].

Since the current study did not undertake phylogenetic analysis, it is not possible to be absolutely certain that the described assault on an HIV-positive male was the source of this individual's infection. However, given that blood contact resulting from violence has previously been documented as a route of HIV transmission, the details of this case do support the conclusion that an assault was very likely the source of infection. Additionally, comparison of data from study records and the qualitative interview revealed a high level of consistency in reported behavior and agreement between data sources.

## Authors' contributions

WS conducted the analyses of the interview data and prepared the first draft of the article. All authors contributed to the design of the study as well to the drafting and revision of the manuscript. All authors have approved the final manuscript.
